# Recommendations from Multi-Disciplinary Professionals for Survivor-Informed and Comprehensive Care for Human Trafficking Survivors

**DOI:** 10.3390/healthcare13233070

**Published:** 2025-11-26

**Authors:** Christina M. Tsoi, Timothy B. Smith, Elizabeth Cutrer-Párraga, Devan Clayton, Joshua M. Marshall, Jamila Mastny

**Affiliations:** Department of Counseling Psychology and Special Education, Brigham Young University, Provo, UT 84602, USA

**Keywords:** community medicine, human trafficking, interdisciplinary communication, mental disorders, mental health services, patient-centered care, psychological trauma, psychotherapy, psychopathology

## Abstract

**Background:** Human trafficking affects millions of people worldwide with multiple adverse outcomes including psychopathology. Although research on human trafficking has become abundant in other academic disciplines (e.g., public health, criminology, social work), healthcare research specific to the mental health treatment of survivors remains limited. **Objective**: The purpose of this study was to gather recommendations from professionals about mental health treatment of trafficking survivors. **Method**: Semi-structured interviews were conducted with 21 multidisciplinary professionals working with trafficking survivors. Data were analyzed using qualitative content analysis methodology. **Results:** An overarching theme derived from the data concerned the recommendation to implement trafficking survivor-informed care, specifically addressing complex trauma and individual client contexts, such as culture and history prior to trafficking. A second theme emphasized the recommendation for comprehensive care, achieved through outreach efforts, interdisciplinary services, case management, and ongoing training for mental health professionals. **Conclusions:** Professionals working with trafficking survivors perceived conventional service formats as insufficient, and they recommended personalized and comprehensive healthcare to address multiple needs and extensive trauma history.

## 1. Introduction

Human trafficking involves the exploitation of persons by means of threat, force, or coercion [1,2]. Individuals vulnerable to entering trafficking situations tend to have prior adverse childhood experiences, including histories of physical and emotional abuse, neglect, or homelessness [3,4,5]. The intensive and ongoing trauma from being trafficked, interacting with a prior history of trauma, results in complex trauma profiles, often characterized by multiple physical and mental health conditions in an entangled web of overlapping symptomatology that complicate healthcare [6].

Psychopathology among trafficking survivors includes combinations of post-traumatic stress disorder, anxiety, and depression [2,5,6]. Suicidal ideation [5,7,8], bipolar disorder, psychoses, conduct disorder, and dissociative disorders are also prevalent [6]. Many survivors further struggle with substance abuse [8], emotional numbing and dysregulation [9], attachment issues [5], paranoia [9], and re-traumatization [10]. The pervasive and deep nature of these patterns affects both physical health and rehabilitation, requiring informed sensitivity from healthcare professionals [6,11].

Professionals agree that interventions with trafficking survivors should be informed by principles of trauma-informed care [11,12]. Trauma-informed care entails principles based on neuroscience research applied to therapeutic relationships, treatments, and survivor empowerment [13,14]. Although trauma-informed care reduces PTSD symptoms and improves recovery among trafficking survivors [12], trauma-informed guidelines can be broad and were not specifically developed for trafficking survivors. The healthcare field needs additional information about effective treatments for survivors of human trafficking [2,15,16].

Understanding the shortcomings of medical and mental health services with human trafficking survivors remains an important aim for research. First, major distinctions between survivors of human trafficking and survivors of other traumas must be made. Beyond common event-specific trauma, human trafficking often entails combinations and iterations of extensive psychological and physical traumas. Trafficking survivors experience physical injuries, intentional manipulation, unpredictable relocation and disorientation, and prolonged and pervasive exploitation involving multiple methods targeting individuals’ vulnerabilities [8,17,18]. Traffickers groom and often normalize the trafficking experience, while simultaneously monitoring and keeping trafficked individuals from information and resources necessary to pursue help [19]. Thus, the loss of control and the complex trauma experienced by human trafficking survivors make it difficult for survivors to plan, trust others, and reconstruct their lives [8]. Amid such disruption and unpredictability, obtaining and completing treatment becomes extremely difficult or a secondary consideration to survival [11].

Nevertheless, while being trafficked, survivors commonly experience evaluations for injury, disease, or psychopathology that put them in touch with healthcare systems and mental health providers [15,20,21,22]. For instance, people being trafficked for sex commonly seek medical treatment for urinary and sexually transmitted infections, violent injuries, and pelvic inflammatory disease. Despite the frequency of interactions with healthcare personnel, many survivors remain undetected [23] or do not receive inquiries or offers for treatment beyond the immediate physical symptom presentation [8]. These important oversights may reflect training and healthcare system deficiencies relevant to human trafficking survivors.

Prior research indicates a lack of instruction and experience working with survivors of human trafficking among medical and mental health professionals [24,25]. Professionals are often unfamiliar with resources relevant to trafficking [13,26]. Furthermore, because the needs of survivors overlap across disciplines [13,27], meeting survivor needs requires carefully coordinated and comprehensive treatment services [16]. Unfortunately, such inclusive care and the associated literature for addressing the complex healthcare needs of trafficking survivors remain limited [1,8,28]. Additional investigation into service provision can yield specific recommendations to better support survivors’ multiple needs.

The purpose of this study was to gather mental health treatment recommendations from multidisciplinary professionals working with survivors of human trafficking. The choice to interview professionals, rather than survivors, was carefully made for several reasons. Since it is difficult to avoid re-traumatizing survivors in research [29], we sought to minimize harm by learning from a variety of professionals who work with them. Moreover, professionals experience a variety of cases over time, which enables them to account for various types of human trafficking, conceptualize issues broadly, and also discern specific trends [30]. In contrast, individual survivors’ trafficking histories vary considerably, with their own intense personal experiences being at the forefront [31]. Thus, learning from the accumulated experiences of professionals working with trafficking survivors avoided the possibility of survivor re-traumatization, diversified the current literature regarding trafficking forms and rehabilitation, and facilitated broad conceptual analyses of the trends and barriers experienced throughout survivor mental health treatment. We employed a descriptive qualitative design with content analysis to examine the perspectives of multidisciplinary participants working to support survivors of human trafficking [32,33]. This approach suited our goal of gathering detailed descriptions about their experiences with survivors [34,35].

The research questions for this study were:

1. What recommendations for improving mental health treatments with trafficking survivors are provided by multidisciplinary professionals working with them?
2. How do multidisciplinary professionals perceive the mental health treatment process for trafficking survivors?

## 2. Materials and Methods

### 2.1. Study Design and Theoretical Framework

We utilized an interpretivist qualitative descriptive framework for this study, which prioritizes understanding participants’ lived experiences and the meanings they ascribe to phenomena within their specific contexts. We received institutional review board approval from our sponsoring University (#2019400) prior to participant recruitment. All participants provided written informed consent before participating.

### 2.2. Participants

The study included 21 professionals (18 female, 3 male) currently working in anti-trafficking organizations that provided survivor rehabilitation. The participants included professionals in various positions in the organizations to gather comprehensive data about the treatment provided to survivors. As such, the sample comprised seven directors of survivor treatment programs, two legal professionals (one specialist, one lawyer), one administrator, two advocates, four case workers, one sexual assault specialist, two basic needs coordinators, one mental health psychiatric nurse, and one trauma specialist. The participants were all familiar with mental health issues of survivors and had provided relevant professional services to survivors from 4 to more than 20 years, allowing them to reflect upon and analyze trends across cases relevant to mental health services and healthcare.

### 2.3. Sample Size

Sample size was determined using Malterud and colleagues’ guidelines [36, considering study aim, sample specificity, theoretical framework, and dialog quality. This study had a focused aim that narrowed the scope of the interviews [36,37]. The specificity of experiences, knowledge, and recommendations from the participants were dense and specific to the target group of professionals directly working with trafficking survivors [36]. The theories used in this study, postpositivism and content analysis, were well researched prior to data collection and analysis [38,39]. In addition, the study was designed to derive results from individual interviews, rather than longitudinal, pre-intervention, and post-intervention studies that require larger sample sizes [37]. Considering all these factors, a sample size of 21 was deemed appropriate for this study [36,37].

### 2.4. Recruitment

Participants were recruited through website searches of anti-trafficking organizations and professional referrals, primarily via email with follow-up phone calls when necessary. The rich descriptions provided by participants demonstrated strong data quality [36,37], and their specific experiences aligned with the target population [38,39]. All participation was voluntary. Although we did not track initial refusals to email solicitations, after individuals agreed to participate, there were no withdrawals from the study.

### 2.5. Data Collection

#### 2.5.1. Interviews

Semi-structured interviews were chosen as the primary data collection method, as they allowed for in-depth exploration of our participants’ complex experiences while maintaining consistent inquiry across participants [40,41]. This approach was particularly appropriate for the sensitive topic of human trafficking, requiring flexibility in questioning and relationship building [42].

Since participants were geographically dispersed across North America, interviews were conducted via Zoom (audio only) or telephone, based on the preference of the interviewee. Zoom interviews were conducted without video to maintain consistency across participants. Interviews lasted approximately 40 min with a 10 min extension if participants requested to continue. Our interview approach offered participants flexibility while maintaining data quality and reducing barriers to participation [43].

All interviews were audio-recorded with consent and conducted in English, with only the participants and interviewers present. Interviews were conducted by trained undergraduate research assistants under lead researchers’ oversight. Training consisted of six hours of initial instruction covering: (a) qualitative interviewing fundamentals, including the purpose and types of interviews; (b) essential interviewer skills such as maintaining neutral affect, active listening, and adapting questions using probes; (c) recognizing and avoiding bias in question formulation and delivery; (d) managing emotional content and identifying nonverbal cues; and (e) post-interview procedures, including reflective note-taking and data security protocols. Training materials emphasized culturally responsive practices and attention to power dynamics in the interview relationship.

To ensure competency and consistency, research assistants participated in mock interviews with senior research team members prior to conducting actual interviews. These practice sessions allowed for real-time feedback on interviewing techniques, question delivery, and probe utilization. Throughout data collection, the lead researchers directly observed and provided feedback on 25% of interviews to monitor fidelity to the interview protocol. The research team held weekly meetings that served multiple purposes: calibration discussions to ensure consistency across interviewers, structured debriefing sessions for research assistants to process emotionally difficult content and address vicarious trauma, and ongoing consultation regarding challenging participant disclosures or interview situations. These safeguards ensured both the quality of data collection and the wellbeing of the research team throughout the study.

Interviews were transcribed verbatim, assigned unique ID numbers, and stripped of identifying information to maintain participant anonymity. Interview recordings and transcripts were stored in password-protected cloud storage, with access restricted to project researchers. Other than spreadsheets to track participants interviewed and coding/themes, no software or AI was used for analyses. The pilot-tested interview questions are provided in Appendix A.

#### 2.5.2. Analytic Memos

After each interview, researchers wrote analytic memos to document emerging ideas, methodological insights, and preliminary interpretations [44]. These memos served to track the analytical process and distinguish novel findings from those supporting existing literature.

### 2.6. Data Analysis

This study used qualitative content analysis to examine both explicit (manifest) and implicit (latent) meanings in participant responses [39,45]. Given the sensitive nature of mental health treatment among trafficking survivors, we adopted an inductive approach, allowing themes to emerge naturally from the data rather than imposing predetermined frameworks [46]. See Table 1 for coding examples. Following Bengtsson’s four-stage process [47], analysis involved:

1. Decontextualization—Transcripts were segmented into meaning units and coded for manifest and, where applicable, latent content, with team discussions guiding interpretation of underlying meanings [47].
2. Recontextualization—Codes were iteratively refined to ensure alignment with participant narratives and comprehensive data coverage [48].
3. Categorization—Codes were grouped into broad categories and then synthesized into overarching themes and subthemes, revealing recurring patterns and deeper insights [45,49].
4. Compilation—Themes and conclusions were validated against the raw data to ensure analytic accuracy [49].

### 2.7. Trustworthiness

We ensured trustworthiness by addressing five key criteria: credibility, dependability, confirmability, authenticity, and transferability [50], guided by the COREQ checklist for transparent reporting [51]. The COREQ Checklist can be referenced in Appendix B. Credibility was meant to be supported through member checking by participants reviewing transcripts and summaries to inform our analysis [47]. Unfortunately, we received no follow-up responses from participants, negating the anticipated benefits of the member checking step. Dependability was strengthened through detailed audit trails [52], analyst triangulation of two to three research team members reviewing the data and analysis [47], and ongoing team meetings to ensure consistent coding and interpretations [50,53].

Confirmability was enhanced through reflexive journaling, allowing our interdisciplinary team to examine how professional backgrounds influenced interpretations while remaining grounded in participant data [54,55,56,57]. To support authenticity, we recruited a diverse sample of practitioners to reflect a range of perspectives on survivor care [50]. Transferability was addressed through thick descriptions of participant roles, service contexts, and the specific circumstances shaping their experiences, enabling readers to assess the applicability of findings to their own settings based on contextual similarities [50,58,59].

### 2.8. Researcher Positionality and Reflexivity

The research team included PhD-level researchers (one female, one male) with more than 30 years of combined expertise in qualitative methods, psychology, mental health psychotherapy (including trauma), and international experience with human trafficking, along with three doctoral students in counseling psychology (two females, one male) and two undergraduate psychology majors (one female, one male). Collectively, the team brought varied levels of clinical experience, research training, and familiarity with trauma-informed practice, positioning members along a continuum from emerging scholars to seasoned experts. No team members had personal experience with being trafficked.

We employed multiple reflexivity strategies to acknowledge our influence on the research process. Interviewers maintained reflexive journals documenting their reactions and assumptions following each interview. Weekly team meetings included structured reflexive discussions examining how our backgrounds, training levels, and prior experiences with trauma-related topics might shape data collection and interpretation. Prior to analysis, team members documented their preconceptions about human trafficking experiences and regularly revisited these assumptions during coding to practice reflexive bracketing. These strategies helped us remain critically aware of our interpretive influence while centering participants’ perspectives and experiences, thereby strengthening the credibility and trustworthiness of our findings.

Furthermore, information power and saturation were assessed according to six dimensions: (a) clearly defined and narrow aim of this study investigating recommendations of mental health treatment for trafficking survivors, (b) sample specificity including only participants currently working in anti-human trafficking organizations and providing direct services to survivors, (c) theoretical grounding in postpositivist and trauma-informed frameworks, (d) the depth, openness, and semi-structure of dialog during interviews, (e) detailed, iterative data analysis, and (f) saturation decisions were verified through coder consensus when discrepancies arose. These seven dimensions were selected because they capture the key indicators of information richness, relevance, and analytic rigor in qualitative research. These criteria provide a systematic basis for determining when sufficient information power and thematic saturation were achieved, ensuring that findings are both well-developed and trustworthy.

## 3. Results

Analysis of participant responses regarding their perceptions of mental health treatments yielded two overarching themes, as shown in Table 2 below. First, participants consistently reported a desire for service providers to take a survivor-centered approach that addresses complex, multilayered traumas and to consider the unique factors that influence survivor experiences. Second, the findings revealed significant gaps in comprehensive care delivery, with participants describing fragmented services that failed to address the full spectrum of survivors’ physical, psychological, and social needs.

### 3.1. Overarching Theme A: Trafficking Survivor-Informed Healthcare

Participants emphasized that while trauma-informed care is helpful, it often fails to meet the complex needs of trafficking survivors. They advocated for survivor-informed care, which centers on restoring autonomy, building trust, and respecting each individual’s readiness and recovery process. As one participant explained, “When you’ve been trafficked you’ve been stripped of your right to make any of your own decisions… client-centered practice… puts all of the decision-making capacity and power back into the client’s hands” [1AS]. Three subthemes illustrate how survivor-informed care must be adapted: (1) a survivor-centered paradigm, (2) addressing multilayered trauma, and (3) ensuring cultural inclusivity.

#### 3.1.1. Subtheme 1: Survivor-Centered Care Enables Recovery

Participants consistently described the need for individualized, survivor-directed treatment. Survivors should guide their own healing journey, from goal-setting to pacing. “People who have been trafficked or tortured have in most cases been completely wiped of their sense of agency. You can help someone make sense of the trauma… but if you are not trying to assist them in regaining the sense of power and sense of control of their life, they are not going to make it very far” [2AS].

Participants repeatedly described survivors as needing to direct their recovery journey and treatment planning. Another participant added, “The client is the expert in their own life… clients know best how they can engage in feasible, practical safety planning, they know what they need to engage in in terms of increasing their economic livelihood, they know what they need to do to best protect their rights in other areas…it would be impossible for us to prioritize the importance of any given focus that our client has” [1AS].

Strong therapeutic relationships are key, but many professionals lack confidence in tailoring care to survivors. Participants highlighted the importance of managing therapist reactions, avoiding rigid symptom focus, and maintaining realistic expectations. “You can never, ever let someone see that you’re shocked by something, because the minute you do… people will start to shut down because they don’t feel like you’re strong enough as a therapist or service provider to handle stuff so they won’t tell you anymore.” [13AS].

Survivor engagement also hinges on flexible treatment structures. Participants criticized rigid timelines and punitive no-show policies, noting that readiness often evolves. “We might have trafficking victims that come to us and their trafficking was very recent … not ready to engage in the mental health piece because they can’t address the trauma… and then we have clients… ten years after their trafficking… ready to now address the trauma… do a deeper dive through mental health treatment” [1AS]. Another stressed, “The profession needs to do a better job of not imposing… letting people determine their own course and respecting their decisions” [5AS].

Participants recommended alternative settings and modalities (e.g., outdoor walking sessions, teletherapy), survivor feedback in program design, and empowering language: “Folks do not identify as victims, they don’t see themselves as victims, and they don’t feel like they’re victims, and so when we’re talking about victim services and wanting to provide victims with health care the stigma is like well you know I’m not a victim so that’s obviously not for me.” [8AS]

#### 3.1.2. Subtheme 2: Multilayered Trauma Requires Holistic Approaches

Participants shared that survivors of trafficking experience layered trauma—before, during, and after exploitation. Many come from abusive or neglectful environments, compounding their exploitation. As one participant explained: “Treatment for human trafficking is treating so many different layers of trauma… experienced through the exploitation or even the trauma that led them to being a human trafficking victim. Because a lot of the survivors… it wasn’t just human trafficking. A lot of them come from homes where they were neglected, abused, sexually abused.” [4AS] A key concept was that the trafficking typically occurred after a history of repeated failures by family or social networks to nurture, protect, or meet emotional needs. It was as if the person had been dropped by the support networks of multiple systems.

Participants emphasized the need for a holistic, trauma-informed approach that acknowledges all stages of trauma. Ignoring this broader context risks ineffective care: “…we can do those things [support with housing, food, work, education, care]… but if that trauma is still there, it’s going to keep causing like reenactments in your life until you address the root causes…” [1AS].

Participants discussed that when survivors contextualize current behaviors within their life history, they gain insight and better manage trauma. They suggested discussing pre-trafficking vulnerabilities—such as domestic violence or homelessness—reduces shame and reinforces the recognition of intentional exploitation by traffickers.

Participants also called for space for survivors to iteratively process the trauma endured during trafficking. Responsive, step-by-step counseling was viewed as critical to prevent re-traumatization. One participant advised: “Sharing trafficking details should happen only when survivors feel safe and ready—probing too early may exacerbate wounds and disrupt engagement.”

Participants recognized that survivors’ relapsing into the trafficking lifestyle is common and therefore requires people intimately familiar with “the life” of trafficking to bridge the gap to mainstream socialization. They specifically suggested involving peer mentoring by post-rehabilitation survivors. Individuals who become peer mentors not only bond with recent survivors but also serve as advocates for systems change: “Getting [survivors] involved in policy decisions…so they can help make these [anti-trafficking] decisions…is a really effective way of keeping the folks out of dangerous lifestyles.” [8AS]

According to participants, survivors commonly use multiple substances to cope and often experience substance dependence. They suggested integrated care was essential, with treatment addressing the intersection of trauma and substance use: “With a substance abuse disorder…I think [therapists] being able to provide support for both…and not just focused on the trauma as separate from the substance abuse.” [5MS]

Participants encouraged other professionals to nurture survivor resilience and hope. In their experience, validating survivors’ strengths helps reduce shame and build motivation for the hard work of transitioning to a better future: “Look, you survived this. You did really well in this situation…you were able to handle a trafficker…so how would you handle an angry customer?” [13AS]. Still, participants repeated that resilience training alone is not enough. Participants stressed the importance of building multiple networks to help survivors prepare for change and reinforce internal motivation with practical and emotional supports essential to long-term survivor recovery.

#### 3.1.3. Subtheme 3: Cultural Competency and Inclusive Access

Participants emphasized the urgent need for culturally responsive and inclusive mental healthcare for trafficking survivors. They stressed that effective treatment must consider survivors’ age, gender identity, sexual orientation, race, ethnicity, and cultural context. Participants reported a major service gap exists for LGBTQ+ survivors, who can experience suboptimal treatment in heteronormative service programs. According to participants, male survivors also encounter disbelief and a lack of tailored services. “We just had a male trafficking survivor… kept in a labor trafficking scenario… but there was no [therapy] group… for him to express that” [10MS].

Participants called for increased cultural competence among providers. They recommended hiring bilingual staff, using trained interpreters, and collaborating with cultural brokers to improve access and outreach. Some programs were recognized for successfully integrating traditional healing methods, such as Native American practices for Indigenous survivors.

Historical trauma was also seen as essential to address. Participants urged providers to consider how collective histories—like slavery or segregation—shape the experiences and recovery of marginalized survivors. Therapist cultural humility and competence were deemed essential. Participants urged hiring bilingual providers, collaborating with cultural brokers, and integrating traditional healing practices where relevant. Participants called attention to historical trauma, particularly in marginalized communities: “What we’re looking at in the African American community is not necessarily the same thing that will work for the Hispanic community, the Asian community, or the Indian community or the Native Americans. … you just have to take that into cultural consideration” [8AS].

### 3.2. Overarching Theme B: Comprehensive Care

Participants emphasized that comprehensive care requires collaboration among systems—law enforcement, mental health, medical providers, community agencies, and more—to support survivors’ long-term recovery. They described key aspects such as interdisciplinary coordination, case management, basic needs provision, housing, and social support. An underlying principle was that since the individuals’ history prior to and during trafficking entailed multiple failures by support networks, effective solutions necessarily involve a network approach rather than a traditional piecemeal, uncoordinated approach.

#### 3.2.1. Subtheme 1: Interdisciplinary Collaboration and Services

Participants spotlighted the importance of coordinated efforts among anti-trafficking and community service organizations to expand access and streamline referrals. One participant explained: “On of the things we do is to ensure that we work in a collaborative manner in our county. So, everybody knows who we are and what the services we provide, and I can be a point of contact to government agencies or nongovernment organizations beneath that work with human trafficking victims.” [4AS]

Participants stressed that different disciplines must learn to communicate effectively:

“I’m an advocate of interdisciplinary conversations and collaborations because we all speak different languages. Social workers have to be able to talk to lawyers, [who] have to be able to talk to doctors, [who] have to be able to talk to the courts and the police. We’ve got to work together.” [10MS]

Though centralized services would be ideal, participants noted they are rare due to funding constraints. Instead, partnering with multiple service sectors—including legal, vocational, medical, and housing—ensures survivors access what they need, even when they live far away.

Participants emphasized the importance of meeting basic needs—like food, safety, and medical care—before expecting engagement in mental health treatment:

“If some of those basic needs are not being met, then they are not able to be in the space to work and engage with higher level [mental health] treatment…” [5MS]. They recommended offering drop-in services (food, hygiene, supplies) for those in transition or not yet able to leave trafficking.

##### Housing

Participants identified safe, stable housing as foundational. One stated:

“The number one barrier that I have found relates to housing. Safe housing. Safe, affordable… long-term, sustainable housing, that does not exist for my clients.” [2MS]

Due to limited short-term options, participants explained that some of their organizations provide initial housing based on assessments of basic and mental health needs. Participants also reported a dearth of long-term housing services that would accept survivors without documentation, such as birth certificates or IDs, which many survivors do not have upon leaving their trafficking situations. One participant suggested that anti-trafficking organizations partner with housing developments to designate transitional, survivor-only units, which can provide housing to survivors while they obtain documentation.

Shelters, participants noted, can be dangerous and retraumatize survivors. For instance, shelter Entry screenings often include invasive questions, and survivors may be turned away after disclosing traumatic experiences. For instance, some housing services will not inform survivors if they have space for them until they screen for qualification. Invasive questions can trigger trauma, compounded when denied services after divulging information. Furthermore, survivors who are offered space in shelters are at increased risk of being dismissed, feeling unsafe, or falling back into the trafficking lifestyle. Participants also described how shelters can cause further harm when survivors see familiar faces from trafficking situations or feel unsafe among others with unstable behavior.

##### Support Systems and Networks

Participants said survivors often exit trafficking alone and in isolation. They stressed the need to help survivors build social support networks for healing and long-term recovery: “Recovery is an ongoing process… developing the social supports that can help you as you meet those barriers or hurdles are important.” [10MS]

Participants indicated that connecting or reconnecting with family members provides the most consistent support to survivors. Participants highlighted that mental health professionals can help with reconciliation when reuniting family members and noted that family therapy can rebuild bonds while members learn to assist one another in recovery. However, some described barriers when families were unwilling or uninvolved:

“Some of the cases… had a parent… who was not as fully engaged… they don’t want us to talk about it… so I think some of my biggest barriers have to do with working with children who have parents who are not fully engaged.” [2MS]

Participants also mentioned the role of community groups, such as churches, especially in African American communities: “Just because the Church is such a big part of so many lives in the African American community… building that community up around people to feel a lot more accepting and a lot more safe.” [8MS]

##### Support for Youth and Foster Care

Participants bemoaned a lack of adequate guardianship and foster services for youth survivors. Participants explained that youth involved in trafficking often experienced inadequate family support, had run away or faced instability in the foster care system, turning to shelters or group homes that exacerbated trauma. Recommendations included efforts to educate foster parents on trafficking warning signs—such as absences or changes in appearance.

Others referenced models like “therapeutic foster home systems,” where caregivers receive training in trauma and trafficking. Participants stressed the importance of equipping all adults in a survivor’s life to recognize trauma responses and support healing.

#### 3.2.2. Subtheme 2: Case Management and Survivor Advocates

Participants consistently emphasized the importance of strong case management to coordinate services and support survivor recovery. Case managers were seen as vital in conducting needs assessments, making referrals, managing logistics, and collaborating with professionals such as educators, medical staff, and legal representatives. As one participant described: “We gain a multi-disciplinary staffing with the case manager, therapist, foster parent, anyone who may be involved in the case including guardians and probation officers…and discuss what would be the best plan of services for the child depending on their needs.” [4AS]

Despite existing partnerships across organizations, participants identified a gap in unified coordination: “There are about 74 organizations… [but] there is no unifying case management system that allows everyone to check in and see what’s going on.” [10MS]

Participants stressed that case management must remain client-centered. Survivors should guide the direction of their care plans. One participant shared: “Whatever the client wants in terms of dictating their own case management plan…we try to keep everything as client-centered as possible… if we had a client who’s housing isn’t secure…and all of their needs is just spent with transportation and purchasing personal care items and they also have a criminal conviction they’re trying to vacate and they also begin engaging in sex work in order to raise money. Many of those things for us might be a given priority but it really depends on the client… [and what they] want to focus on first.” [1AS]

In addition to case managers, participants articulated the crucial role of survivor advocates—individuals with lived experience who return to support others exiting trafficking. They explained that survivor advocates often serve as the first point of contact and meet survivors wherever they are—hotels, police stations, or street corners. They explained further that survivor advocates are essential in helping survivors build trust with the treatment process, as they can relate to them first-hand. As one participant explained: “Support for these victims is incredible when they have a prior survivor saying, ‘I’ve been where you are, and I’m going to walk his road with you or help you or just here are the resources,’ it’s a powerful thing.” [8MS]

#### 3.2.3. Subtheme 3: Interdisciplinary Training

Participants emphasized that many mental health professionals lack sufficient training on human trafficking and trauma-informed care. They stressed the need for interdisciplinary education that includes not only therapists, but also all staff members who interact with survivors—such as receptionists and support personnel.

One participant urged comprehensive training across sectors: “We have to train all levels of providers. We have to train them that this is real, it is not just a niche topic… we must train healthcare, we must train providers, we must train anyone doing mental health treatments… Education. Education. Awareness.” [8MS]

Participants suggested that professionals from mental health, law enforcement, legal, and medical fields meet to identify specific training needs and share best practices. They also highly recommended training intake staff to recognize signs of abuse or trafficking and to assess for adverse childhood experiences. Participants shared that misinterpreting survivor behaviors—such as defiance—was a common concern, noting these behaviors often reflect self-protection, not disinterest. Participants highlighted several key training areas:

- Building trust with survivors;
- Working effectively with survivor advocates;
- Integrating substance use treatment;
- Understanding trauma responses.

They also recommended that mental health staff pursue ongoing learning and create space within therapy to learn directly from survivors. Since non-survivors cannot fully grasp the trafficking experience, participants said professionals must remain open, listen actively, and consult with survivors in recovery for guidance.

#### 3.2.4. Subtheme 4: Outreach

##### Outreach as Education and Awareness

Participants consistently emphasized the critical role of outreach in combating human trafficking. They described outreach as essential not only for identifying and connecting survivors to services, but also for raising public awareness, preventing future exploitation, and challenging harmful myths. While one participant noted some success with awareness efforts, most expressed that far more outreach is needed. As one explained, “A lot more education about what trafficking [is], how it walks and talks, what it looks like… like whatever you think trafficking is, it’s far more insidious and more mundane… it’s just happening all around us… if you learn to open your eyes you see it everywhere… a lot more education is necessary about it.” [10AS]

##### Reducing Stigma and Shaping Public Response

Participants believed that outreach helps reduce stigma related to both trafficking and mental health treatment. They believed it also builds momentum for prevention and systemic change. One participant emphasized the need to engage the broader public, stating, “It’s important that we provide services to survivors, but it’s also important that we help educate the general population… the public has to be outraged about it. They have to be on the side of wanting to eliminate it, not harvest an issue.” [9AS]

##### System-Level Engagement

Several participants suggested that outreach must operate across individual, community, and policy levels. They explained that people working directly with survivors must be in conversation with lawmakers and communities to correct misperceptions about what trafficking looks like. One participant noted, “Our big platform is that mezzo, micro, and macro all have to be talking… the micro people… need to be talking to the people who are making the laws… because I think that communities still don’t have a sense of what trafficking actually is… they think it’s this van that comes and whisks people away and puts them in a cage somewhere and just not true for the most part and that tells me that the systems still aren’t talking…. so until we paint the correct picture of who trafficking survivors are, who is being trafficked and how it is impacting communities, simply we’re never going to provide correct services.” [10MS]

##### Targeting High-Risk Environments and Families

Participants recommended expanding outreach in schools, child welfare services, law enforcement, and rehabilitation centers—particularly in regions most impacted by trafficking. They also stressed that outreach must address family systems, where initial trauma often begins. One participant stated, “A lot of sexual assault… a lot of violence… happens at home… Victims… are vulnerable because of [what has] happened in their homes. We haven’t done a good enough job yet in society to really talk about what’s happening in peoples’ homes… How did it get there? What pushes a young person out on the streets to be vulnerable? To go through what they go through and to make the decisions that they make? So those are all areas that I feel are huge barriers in a way to servicing our victims and survivors.” [7AS]

##### Survivor-Led Efforts

Some participants described successful outreach strategies already in place. One shared how an organization trains local volunteers—many of whom are survivors of domestic violence, sexual assault, or trafficking—to become outreach specialists. These volunteers, often bilingual, act as peer educators and serve as liaisons between the agency and the community. They identify local needs and lead prevention campaigns tailored to the populations they serve.

##### Professional Engagement in Outreach

Participants encouraged psychotherapists to engage directly in outreach by offering presentations and trainings on human trafficking, domestic violence, and sexual assault. They suggested school-based programs focused on topics of relationships, consent, and healthy masculinity. One participant referenced a school human trafficking club that sponsors symposiums, conferences, and classes about human trafficking. Additional outreach ideas included bilingual performances based on survivor stories, multilingual public service announcements, and social media awareness campaigns. Participants also recommended simply approaching organizations and asking if they are open to learning more about trafficking. They noted that collecting data on outreach impact could improve future programming and help secure funding.

##### Direct Engagement with Survivors

Finally, participants emphasized the need for targeted outreach to survivors themselves. They advocated for direct engagement in locations such as jails, substance use treatment centers, and on the streets—particularly in areas where traffickers may be active. One participant described this work, stating, “Actually going into the streets, building relationships… getting them to know who we are, trust us… even if it’s just coming to a support group… Through that outreach, we’re able to give them personal hygiene products, condoms, resources, snacks, clean needles, bandages… and stuff that is needed.” [6AS]

### 3.3. Novel Findings

The findings from this study revealed several novel and unexpected insights that expand current understandings of trauma recovery and service delivery for trafficking survivors. Participants drew a distinction between trauma-informed and survivor-informed care, emphasizing the need to prioritize survivor autonomy, decision-making, and control—an approach seldom operationalized in practice. They also underscored the importance of addressing trauma as cumulative, spanning pre-, during-, and post-trafficking experiences, and advocated for flexible, survivor-paced engagement models rather than rigid clinical structures. Cultural adaptation—not just competency—was framed as essential, with calls to integrate historical trauma and culturally specific healing practices into care. Survivor advocates were described not as supportive extras, but as central connectors to care, trust, and safety. Furthermore, outreach was reframed as a clinical intervention rather than solely a public awareness strategy, with participants describing direct engagement in high-risk settings as critical to service access. Lastly, participants envisioned care coordination at a systems level, highlighting the need for shared case management tools and structural alignment between service providers, policy makers, and community stakeholders. Overall, these recommendations invite a shift from stand-alone mental health service provision to a network approach in which mental healthcare is integrated across services through effective coordination and case management. Thus, these findings offer concrete and novel directions for transforming how services for trafficking survivors are conceptualized and delivered (see Table 3).

## 4. Discussion

Human trafficking results in serious mental health consequences for survivors. Although many organizations worldwide have emerged to promote survivors’ recovery, concerns remain about inadequate mental health treatment. To address that shortcoming, this study gathered treatment recommendations from multidisciplinary professionals working with survivors of human trafficking to inform mental health providers and healthcare systems.

In response to our first research question about professionals’ recommendations for improving mental health treatments with trafficking survivors, the multidisciplinary interviewees tended to emphasize shifting from traditional models of weekly individual psychotherapy to flexible survivor-informed care. While abundant prior literature has discussed the importance of trauma-informed care [11,25,60,61,62], the professionals interviewed emphasized the need to shift from generic trauma-informed counseling to radically personalized treatment [12].

Trauma-informed care is widely recognized as a framework that organizes trauma recovery services around the core principles of safety, trust, choice, collaboration, and empowerment, as outlined in frameworks such as the SAMHSA trauma-informed principles and ecological models of care [11,25,60,61,62]. Our data indicated the need for a trafficking-specific treatment approach that goes beyond trauma-informed care principles.

Survivor-informed care is defined as an approach to service delivery that integrates the lived expertise, agency, and priorities of trafficking survivors into every level of their treatment process, akin to the concepts of patient-centered care [63] and patient empowerment [64]. Specifically, survivor-informed care transfers procedural control to survivors (e.g., timing, length, location, and attendance requirements of treatment), explicitly addresses multilayered trauma (pre-, peri-, and post-trafficking, including trauma-bonding and substance use), and redesigns access around real-world barriers (cost/eligibility, insurance, immigration fear, language, housing, transportation) through comprehensive, interdisciplinary services. Participants consistently emphasized this survivor-led orientation, describing the importance of autonomy and collaboration in treatment decisions (e.g., “…letting people determine their own course…” [5AS]; “…depends on the client…we work in partnership” [1AS]). Survivor-informed care operationalizes trauma-informed principles for trafficking contexts by emphasizing concrete adaptations, such as flexible attendance options, drop-in supports, bilingual and survivor-advocate staffing, outreach efforts, and the inclusion of cultural brokers, all of which have rarely been examined in mental health research.

As has been emphasized by prior research on patient-centered care, such an approach denotes a fundamental shift in how healthcare is conceptualized and delivered [65]. The professionals affirming this approach in our interviews conceptualized survivors as experts in their own healing, trusting them to know what they need at each step of their recovery process. The professionals emphasized many benefits from empowering survivors to direct every aspect of their healing process, not just what happens in the therapy room [64]. Professionals adjust to survivor feedback, use terminology congruent with survivor lingo, and align with the survivor to reestablish and reframe trusting relationships through corrective experiences and meaningful interdependence. The genuine compassion and decision-making support characteristic of patient-centered care and empowerment were deemed essential by those we interviewed yet have been shown to be lacking in contemporary healthcare contexts [63].

The interviewees were critical of existing healthcare systems. The multifaceted nature of trafficking survivor trauma can be overlooked in typical medical evaluations and mental health counseling [8]. Professionals commonly focus solely on the trauma experienced during trafficking, but neglect addressing trauma encountered pre- and post-trafficking [3,4]. It is imperative that professionals make room for survivors to process all aspects of their trauma, including adverse childhood experiences, historical trauma, multigenerational trauma, trauma from trafficking, substance use, and relapsing back into the trafficking lifestyle [7]. Participants elaborated that survivor traumas can layer and intertwine. They explained that if left unprocessed, these multifaceted traumas often exacerbate the hurdles faced throughout treatment. Therefore, addressing all aspects of the survivors’ history can reduce the likelihood of future trauma. It can also decrease shame, cultivate autonomy, and foster empowerment to help them move toward the lives they hope for.

Healthcare professionals benefit from acknowledging the unique multicultural background, experiences, and demographics of each survivor [65]. This includes aligning with the experiences of survivors of various gender identities and sexual orientations [13,66]. Participants elaborated on the need to address specific contextual factors, such as toxic masculinity and power differences across gender, and dynamics intersecting with LGBTQ+ experiences. To improve culturally sensitive treatments, interviewees recommended the recruitment of bilingual professionals and the involvement of cultural brokers or traditional healers in the treatment process.

Regarding our second research question about multidisciplinary professionals’ perception of the mental health treatment process for trafficking survivors, professionals strongly emphasized interdisciplinary cooperation and comprehensive care [25,61,67]. Many different professional disciplines encounter survivors [68,69]. However, our participants affirmed that coordination is insufficient and haphazard [61,70], failing to provide comprehensive care to addresses the immediate (e.g., emergency services), ongoing (e.g., mental health, substance abuse), and long-term (e.g., life skills training) needs of survivors [70]. Participants pled for basic living skills [5,71], multi-disciplinary service provision [25,27], strengthening existing social circles, and community integration [72]. For instance, many survivors exit trafficking without any safe place to live [70,73], with teens facing major impediments to education, such as removal from school [74] and developmental delays [72]. Furthermore, social isolation is the norm [71,75], such that typical one-on-one treatments for mental health are entirely insufficient to meet survivor mental health needs [69,76]. Coordinated comprehensive care helps to identify survivors and address their multiple concerns [2,66,77], such as secure housing [78] and social supports, and foster care placement training and improvements.

Multidisciplinary professionals emphasized that mental health providers should not merely treat symptoms but also help survivors expand and strengthen their personal support networks. When survivors exit their trafficking situations without a support system, establishing or reestablishing strong interpersonal relationships can reduce the risk of relapsing back into the trafficking lifestyle. Specifically, participants suggested therapy involving survivors’ support systems, particularly parents, siblings, and extended relatives. This approach can be balanced with concerns about risks of dysfunctional family dynamics [79,80,81,82], but the emphasis is to repair, rather than repeat problematic dynamics. Consultation with survivors can determine how and when to focus on familial relationships [83].

Strong case management was described as essential in connecting survivors to resources and collaboration between treatment teams. A survivor-centered approach to case management emphasizes survivor autonomy and service prioritization [84,85].

In addition, several participants emphasized the necessity of survivor advocates. Survivor advocates act as liaisons between survivors and organizations or resources, but they are often trafficking survivors themselves. Having personal experiences with both trafficking and ongoing recovery, they can mentor survivors in ways that professionals cannot.

Training for professionals is another key consideration, too often overlooked [24,25,63,86]. Prior research indicates that many healthcare professionals have a surface-level understandings of the nature and consequences of human trafficking [87], which adversely impacts survivors’ experience in treatment [8]. As confirmed by the professionals we interviewed, an accurate understanding of survivors’ context is imperative for effective treatment [86,88].

Survivor experiences vary significantly [62]. Therefore, participants stated that universally applying standardized treatment to all trafficking cases will likely neglect the actual needs of an individual survivor. Emphasizing a survivor-centered approach will likely help survivors with a variety of backgrounds and mental health concerns.

Taking a broad view, the multidisciplinary participants in this study affirmed that healthcare providers working with survivors of human trafficking simply lack the resources to address current needs, let alone to prevent future cases. Focused outreach efforts are therefore needed to increase public awareness of human trafficking and more actively take steps to curtail trafficking. As with comprehensive care in terms of recovery, there is no limit to who can assist with outreach or advocacy efforts. Many participants suggested a collaboration between professionals and the public to ensure that outreach occurs in the education system, legal system, addiction recovery centers, and youth organizations. Outreach efforts can equip individuals with an understanding of their rights and reduce unhelpful stereotypes [19].

### 4.1. Implications for Healthcare Providers and Organizations

Both the current findings and previous research suggest that healthcare professionals benefit from learning more about human trafficking and its impact on survivor mental health [25,50]. In addition to seeking out continuing education, therapists will benefit from collaborating on treatment plans and learning from professionals from other fields also encountering survivors, including social work, criminal justice, and nursing [69]. Professional organizations could publish online resources and designate tracks in conferences that address populations likely to be misunderstood or inappropriately served by traditional mental health services. Professional training programs in psychology and psychiatry can offer courses on comprehensive, trauma-informed care with curricula inclusive of human trafficking considerations. Although graduate programs cannot teach everything, they can support opportunities for student learning, such as through collaborating with other institutions, offering online training, or providing scholarships for students to attend conferences and workshops [89].

Just as professionals benefit from becoming more informed about human trafficking and severe trauma contexts, strong benefits can accrue as professionals share accurate information with community services and public audiences. Mental health professionals can participate in or organize outreach efforts to improve community awareness about the dynamics that maintain and help to reduce the commodification of humans [90]. They can also advocate for survivor client needs, such as in court settings or by informing public policy [91]. Table 4 lists specific implications for professionals.

### 4.2. Limitations and Recommendations for Future Research

This study evaluated the perspectives of multidisciplinary professionals working with survivors. Survivors’ experiences would add depth to the results, but we intentionally avoided interviewing survivors to prevent participant re-traumatization and trend mischaracterization. Nevertheless, future research studies could improve our methods by asking survivors to review summaries of qualitative research themes. Such a review of broad concepts would ostensibly be less likely to trigger trauma than one-on-one, introspective interview questions. Alternative methods to minimize trauma reactions could include screening participants for trauma susceptibility, conducting emotionally supportive focus groups, or soliciting anonymous online responses/journals. However, such volunteer participants may differ in their perspectives from those actively experiencing trauma.

A related consideration is that while this study evaluated observations about treatment improvement, future research will need to directly evaluate any changes in services and outcomes. Mental health treatment delivery and effectiveness may vary from professionals’ expectations. Data explicitly evaluating factors that enhance survivors’ recovery remains indispensable.

The professionals interviewed in this study provided many services to survivors, not solely mental health services. Although this proved advantageous in soliciting diverse perspectives, the participants varied in their depth of knowledge about mental health treatments, such that some spoke more authoritatively than others. While all participants shared information that they had heard from trafficking survivors, those with mental health backgrounds tended to provide more nuanced insights about therapeutic processes. Those with other areas of expertise shared recommendations relevant to their work, such as the need for case management and inter-agency collaboration. The breadth of perspectives can be seen as both a strength and limitation of this study. We encourage future research to evaluate the multi-disciplinary approaches strongly recommended by professionals in this study.

A related limitation was the heterogeneity of populations served by the professionals interviewed. Most of their agencies focused on sex trafficking, but others also addressed labor trafficking. Thus, the results may be more applicable to survivors of sexual exploitation than forced labor. Nevertheless, a strength of this study was that the professionals reported working with survivors with varied levels of trauma and with a broad range of demographic backgrounds. The diversity among the survivors they served was a primary reason for their recommendations to individualize and adapt treatment based on trauma profile and experiences during trafficking, as well as on culture, ethnicity, age, and gender. Future research can consider both population-specific issues and broad approaches that enable agencies to address the wide variety of circumstances and backgrounds among survivors.

As a separate methodological consideration, receiving direct feedback through member checking would have been beneficial. To support credibility, we contacted all participants, asking for their feedback on our interpretation of the data. Unfortunately, we did not receive any responses. This lack of feedback cannot be equated with tacit acceptance of our findings, since the more likely explanation is a lack of interviewee availability to respond to our requests for feedback due to their other professional responsibilities.

The participants in this study worked in North America, so the results are unlikely to generalize to other world regions with different mental health services available. Moreover, data collection occurred at the time when public health mandates to prevent the spread of COVID-19 may have influenced the context of participants’ responses. Their interview responses reflected years of accumulated experience and were not specific to the circumstances of the pandemic. In any case, future research can build on the present study by intentionally addressing the limitations of the present research, which nonetheless offers several contributions to the field.

## 5. Conclusions

Pervasive worldwide, human trafficking leaves survivors with profound mental health consequences, which interact with physical health, vocational rehabilitation, family/societal reintegration, and virtually every other aspect of daily functioning. The multidisciplinary professionals interviewed in this study consistently emphasized taking an empowering survivor-centered approach to treatment that capitalizes on survivor resilience, experience, and strengths. They also urged agencies and healthcare systems to actively coordinate case management to expand the range of available services and to facilitate basic needs, such as housing and safety, simultaneously with healthcare and mental health services. They pointed out that most healthcare systems are not set up in these ways. Therefore, this study calls for systemic redesign of healthcare to meet the intensive but understandable needs of survivors of human trafficking. Simply stated, the cure must be sufficient to the circumstance. The root circumstances causing mental health conditions must be addressed, not merely the symptoms. The key premise is that since human trafficking itself denotes societal failures at multiple levels (child protection, family supports, welfare services, enforcement of existing laws against trafficking, etc.), viable solutions necessarily entail societal investment in multilevel coordinated supports and integration into supportive networks or families. At the very least, mental health services with this population must engage with other agencies and shift from formalized, weekly counseling sessions to collaborative treatments in multiple formats. Countering the dehumanization of human trafficking entails humanization, not merely counseling. A personalized integration of trafficking survivors into supportive social/familial and vocational requires a network model of compassionate care.

## Figures and Tables

**Table 1:** Codebook examples, including data quotation, meaning unit, code, category, and theme.

Raw Data, Quotation	Meaning Unit	Code	Category	Theme
[referring to clients who do not have their basic needs met] “Many of those things for us [mental health providers] might be a given priority, but it really depends on the client who works best in partnerships, saying this is the thing that I want to focus on first, and this is the way that I want to go about it, and based on how the client plays that out, that is how we work in partnership with the client to help support them.” [1AS]	Survivor-led prioritization guides collaborative and comprehensive care	Therapist–Client Interaction	Therapeutic Processes	Current Successful Practices
“Yeah, I think just in general, the profession needs to do a better job of not imposing, you know, it’s this whole self-determination process, kind of letting people determine their own course and respecting their decisions in that. Because not everyone is going to be ready for immediate services.” [5AS]	Professionals should respect client autonomy and readiness and timing for change	Timing of Treatment	Setting of Treatment	Recommendations for Further Practices
“Sometimes this linguistic barrier, so in that area we have several people from the Philippines, and so finding people who are used to working with Tagalog, in-person interpreters, which is kind of a very specific part of mental health, I guess you can say because having an in-person interpreter can be difficult and challenging as far as the session goes.” [5MS]	Language and interpretation barriers to treatment	Race/Culture	Multicultural Considerations	Recommendations for Further Practices

**Table 2:** Healthcare Themes and Illustrative Quotes.

Theme A: Trafficking Survivor-Informed Healthcare	
Subtheme	Illustrative Quote
Subtheme 1: Survivor-Centered Care Enables Recovery	The client is the expert in their own life… clients know best how they can engage in feasible, practical safety planning, they know what they need to engage in in terms of increasing their economic livelihood, they know what they need to do to best protect their rights in other areas…it would be impossible for us to prioritize the importance of any given focus that our client has [1AS]
Subtheme 2: Multifaceted Trauma Requires Holistic Approaches	Treatment for human trafficking is treating so many different layers of trauma… experienced through the exploitation or even the trauma that led them to being a human trafficking victim. Because a lot of the survivors… it wasn’t just human trafficking. A lot of them come from homes where they were neglected, abused, sexually abused… [4AS]
Subtheme 3: Cultural Competency and Inclusive Access	What we’re looking at in the African American community is not necessarily the same thing that will work for the Hispanic community, the Asian community, or the Indian community or the Native Americans. So… for this specific population what can we do and take that into cultural consideration. [8AS]
**Theme B: Comprehensive Care**	
**Subtheme**	**Illustrative Quote**
Subtheme 1: Interdisciplinary Collaboration and Services	I’m an advocate of interdisciplinary conversations and collaborations because we all speak different languages. Social workers have to be able to talk to lawyers, [who] have to be able to talk to doctors, [who] have to be able to talk to the courts and the police. We’ve got to work together.
Subtheme 2: Case Management and Survivor Advocates	There are about 74 organizations… [but] there is no unifying case management system that allows everyone to check in and see what’s going on. [10MS]
Subtheme 3: Interdisciplinary Training	We have to train all levels of providers. We have to train them that this is real, it is not just a niche topic… we must train healthcare, we must train providers, we must train anyone doing mental health treatments… Education. Education. Awareness. [8MS]
Subtheme 4: Outreach	Actually going into the streets, building relationships… getting them to know who we are, trust us… even if it’s just coming to a support group… Through that outreach, we’re able to give them personal hygiene products, condoms, resources, snacks, clean needles, bandages… and stuff that is needed. [6AS]

**Table 3:** Novel and Rare Contributions in the Findings.

Theme	Description and Illustrative Quote
Explicit Distinction Between Trauma-Informed and Survivor-Informed Care	Recommended substantial individualization of treatment beyond trauma considerations to align with patient empowerment conceptualizations of healthcare. “Client-centered practice… puts all of the decision-making capacity and power back into the client’s hands” [1AS].
Integration of Pre-, During-, and Post-Trafficking Trauma as Standard Clinical Framing	Rather than viewing trafficking trauma as isolated, participants stressed treating trauma as cumulative and layered, including family dysfunction, systemic neglect, and exploitation. “Treatment for human trafficking is treating so many different layers of trauma… it wasn’t just human trafficking” [4AS].
Critique of Rigid Clinical Structures and Advocacy for Flexible Engagement Models	Rejection of rigid mental health structures (e.g., no-show penalties, fixed timelines) in favor of flexible treatments including walking sessions, drop-ins, and therapy years after the trafficking event. “We might have trafficking victims…not ready to engage…then we have clients…ten years after…ready now to address the trauma” [1AS].
Culture-Specific Adaptations	Participants emphasized cultural adaptation with tailored care models across racial, ethnic, and LGBTQ+ communities, including collective trauma analysis, religious alignment, and traditional healing practices. “What we’re looking at in the African American community is not necessarily the same thing that will work for the Hispanic…Asian…Native Americans” [8AS].
Survivor Advocates as Essential System Connectors, Not Supplemental Roles	Prior survivors can be foundational to recent survivors’ treatment as advocates and mentors, beyond mere “peer support,” by serving as first contact, crisis responder, treatment facilitator, and interagency liaison. “When they have a prior survivor saying, ‘I’ve been where you are’…it’s a powerful thing” [8MS].
Outreach Framed as Clinical Intervention	Participants positioned outreach (street-based harm reduction, school symposiums, multilingual PSAs, jail engagement) as clinical entry points for healing, reconceptualizing outreach as therapeutic access. “Actually going into the streets, building relationships… trust us…then they want to come in” [6AS].
Systemic Integration Beyond Interdisciplinary Teams	Participants described the need for comprehensive system coordination, including shared databases, inter-agency case management, and macro–micro alignment, pushing toward unified survivor care ecosystems. “There are about 74 organizations…but there is no unifying case management system” [10MS].

Note. Numbers in brackets indicate participant identifiers. Quotes are excerpted from semi-structured interviews conducted with multidisciplinary professionals.

**Table 4:** Implications for Mental Health Treatment for Trafficking Survivors.

Trafficking Survivor-Informed Healthcare
Flexibility, patience, and leniency with treatment logistics. Become familiar with “the life” of trafficking, including associated lingo and experiences to avoid judgmental or shocked reactions. Allow survivors to determine how the treatment process will play out (e.g., timing, length, location, and attendance requirements of treatment), aligning with individuals’ readiness, which can vary each meeting. Seek and incorporate survivor feedback into treatment plans. See survivors as genuine survivors and use terminology congruent with resilience and empowerment.
**Multilayered Trauma**
Explicitly teach survivors about what trauma is and how it can manifest in their life (e.g., dissociation). Explicitly help survivors understand how their pre-trafficking trauma impacted their trafficking trauma. Help survivors understand multifaceted trauma and specifically how it impacts multiple areas of daily life. Address trafficking-specific exploitation and trauma-bonding in iterative steps, not all at once. Address interrelated and overlapping factors like substance use and legal violations. Understand that return to “the life” of trafficking is common, and try to prevent it by establishing stronger social networks, including family reintegration and mentoring from former survivors now experiencing positive functioning. Understand that establishing supportive networks is more beneficial than solely focusing on resilience or cognitive approaches to treatment.
**Multicultural Considerations**
Implement cultural healing strategies into treatment, such as involving local community/religious leaders or traditional healers. Align treatment with survivors’ age, gender identity, sexual orientation, etc., and demonstrate cultural humility.
**Comprehensive Care**
**Interdisciplinary Collaboration and Services**
Develop collaborations across agencies, including data sharing agreements and communication, learning terminology used in other disciplines. Meet basic needs, providing drop-in services where survivors who are unable/unprepared to leave trafficking can obtain food, healthcare, and other essential resources. Establish survivor-only housing that does not mix with other unhoused or psychiatric populations. Educate survivors’ families and extended support networks about trafficking and trauma. For child and youth survivors, develop specialized foster care systems and explicitly inform foster parents about trafficking and trauma.
**Case Management**
Use a survivor-centered approach to case management; survivors identify their own needs. Hire or work with survivor advocates, including recovering survivors, to expedite trust and relationship building with survivors.
**Interdisciplinary Training**
Focus on skills and feedback to trainees. Bring professionals from different fields together and have them train each other on various aspects of how to effectively work with survivors. Train professionals on assessments to identify survivors (e.g., Adverse Childhood Experiences assessment of multiple traumas). Help professionals understand trafficking-specific behaviors that may hinder treatment (e.g., deviance and what it means). Hire survivors as trainers to teach professionals about the trafficking experience. Make training accessible to different disciplines.
**Outreach/Advocacy**
Collaborate with professionals (at all levels) and the public to implement wider outreach efforts. As part of rehabilitation, involve survivors in advocacy and outreach programs, such as reducing stigma and humanizing public perceptions of survivors. Implement school-based prevention addressing topics of relationships, consent, and healthy vs. toxic masculinity. Get outside the office and implement outreach in high-risk communities, jails, and substance use rehabilitation programs to build relationships and educate survivors about mental health resources.

## Data Availability

Due to the sensitive nature of this research and ethical obligations to protect participant confidentiality, interview data are not publicly available. Requests for de-identified data from qualified researchers will be considered on a case-by-case basis, subject to institutional ethics approval, data use agreement, and alignment with original participant consent. Requests should be directed to the corresponding author.

## Abbreviations

The following abbreviations are used in this manuscript:

PTSD	Post-Traumatic Stress Disorder

## Appendix A. Interview Questions

1. Can you briefly tell me about your role in providing treatment for survivors of trafficking?
2. Tell me about your experience providing emotional/mental health treatment/support to survivors.
3. What has surprised you about the treatment process for trafficking survivors?
4. How has your perspective of providing treatment changed the longer you’ve worked with trafficking survivors?
5. What do survivors say about their experience with therapy/counseling?
6. Please think of a mental health treatment “success” case. What factors played a part in that?
7. Now think of a case that you thought was less successful. What factors played a part in that?
8. What have you learned about helping survivors of trafficking that other professionals aren’t talking about that much yet?
9. What recommendations would you give to a therapist trying to help survivors of human trafficking that might go beyond what they already do with other clients?

## Appendix B. COREQ Checklist

Consolidated Criteria for Reporting Qualitative Research

Recommendations for the mental health treatment of human trafficking survivors

Domain 1: Research Team and Reflexivity

Personal Characteristics

**No.**	**Item**	**Guide Questions/Description**	**Reported?**	**Page/Section**
1	Interviewer/facilitator	Which author/s conducted the interview or focus group?	Yes	Methods section, p. 4
2	Credentials	What were the researcher’s credentials? (e.g., PhD, MD)	Yes	Methods section, p. 6
3	Occupation	What was their occupation at the time of the study?	Yes	Methods section, p. 6
4	Gender	Was the researcher male or female?	Yes	Methods section, p. 6
5	Experience and training	What experience or training did the researcher have?	Yes	Methods section, p. 4 & 6

Relationship with Participants

**No.**	**Item**	**Guide Questions/Description**	**Reported?**	**Page/Section**
6	Relationship established	Was a relationship established prior to study commencement?	Yes—no prior relationship	Methods section, p. 4
7	Participant knowledge of the interviewer	What did the participants know about the researcher? (e.g., personal goals, reasons for conducting the research)	Yes	Methods section, p. 3
8	Interviewer characteristics	What characteristics were reported about the interviewer/facilitator? (e.g., bias, assumptions, reasons and interests in the research topic)	Yes	Interviewers’ positionality and reflexivity, p. 6–7

Domain 2: Study Design

Theoretical Framework

**No.**	**Item**	**Guide Questions/Description**	**Reported?**	**Page/Section**
9	Methodological orientation and Theory	What methodological orientation was stated to underpin the study? (e.g., grounded theory, discourse analysis, ethnography, phenomenology, content analysis)	Yes	Methods section, p. 3

Participant Selection

**No.**	**Item**	**Guide Questions/Description**	**Reported?**	**Page/Section**
10	Sampling	How were participants selected? (e.g., purposive, convenience, consecutive, snowball)	Yes	Methods section, p. 3
11	Method of approach	How were participants approached? (e.g., face-to-face, telephone, mail, email)	Yes	Methods section, p. 3
12	Sample size	How many participants were in the study?	Yes	Methods section, p. 3
13	Non-participation	How many people refused to participate or dropped out? Reasons?	No for recruitment; Yes for drop-outs	Methods section, p. 4

Setting

**No.**	**Item**	**Guide Questions/Description**	**Reported?**	**Page/Section**
14	Setting of data collection	Where was the data collected? (e.g., home, clinic, workplace)	Yes	Methods section, p. 4
15	Presence of non-participants	Was anyone else present besides the participants and researchers?	Yes	Methods section (no one else present), p. 4
16	Description of sample	What are the important characteristics of the sample? (e.g., demographic data, date)	Yes	Methods section, p. 3

Data Collection

**No.**	**Item**	**Guide Questions/Description**	**Reported?**	**Page/Section**
17	Interview guide	Were questions, prompts, guides provided by the authors? Was it pilot tested?	Yes	Methods section, p. 4 and Appendix A
18	Repeat interviews	Were repeat interviews carried out? If yes, how many?	N/A	
19	Audio/visual recording	Did the research use audio or visual recording to collect the data?	Yes	Methods section, p. 4
20	Field notes	Were field notes made during and/or after the interview or focus group?	Yes	Method section, analytic memos, p. 5
21	Duration	What was the duration of the interviews or focus group?	Yes	Methods section, p. 4
22	Data saturation	Was data saturation discussed?	Yes	Methods Section, p. 7
23	Transcripts returned	Were transcripts returned to participants for comment and/or correction?	Yes	Methods Section, p. 6 (Although participants were contacted, none responded).

Domain 3: Analysis and Findings

Data Analysis

**No.**	**Item**	**Guide Questions/Description**	**Reported?**	**Page/Section**
24	Number of data coders	How many data coders coded the data?	Yes	Methods section, p. 6
25	Description of the coding tree	Did authors provide a description of the coding tree?	Yes	Methods section, p. 5–6 and Table 1
26	Derivation of themes	Were themes identified in advance or derived from the data?	Yes	Method section, p. 3 and 5 (Derived from data, not in advance)
27	Software	What software, if applicable, was used to manage the data?	Yes	Methods Section, p. 4 (No software used except Word and Excel).
28	Participant checking	Did participants provide feedback on the findings?	Yes	Method Section, p. 6 (We requested feedback on the findings, but no participants responded).

Reporting

**No.**	**Item**	**Guide Questions/Description**	**Reported?**	**Page/Section**
29	Quotations presented	Were participant quotations presented to illustrate the themes/findings? Was each quotation identified? (e.g., participant number)	Yes	Results, p. 7–15 and Table 2 and Table 3
30	Data and findings consistent	Was there consistency between the data presented and the findings?	Yes	Results, p. 7–15 and Table 2 and Table 3
31	Clarity of major themes	Were major themes clearly presented in the findings?	Yes	Results, p. 7–15 and Table 2 and Table 3
32	Clarity of minor themes	Is there a description of diverse cases or discussion of minor themes?	Yes	Results, p. 7–15 subthemes
